# Depictions of Heroism in Battle and Anguish from Tuberculosis

**DOI:** 10.3201/eid2203.AC2203

**Published:** 2016-03

**Authors:** Terence Chorba, Byron Breedlove

**Affiliations:** Centers for Disease Control and Prevention, Atlanta, Georgia, USA

**Keywords:** art science connection, emerging infectious diseases, art and medicine, about the cover, La muerte de Girardot en Bárbula, Cristóbal Rojas, The death of Girardot in Bárbula, depictions of heroism in battle and anguish from tuberculosis, multidrug resistance, tuberculosis, public health

**Figure:**
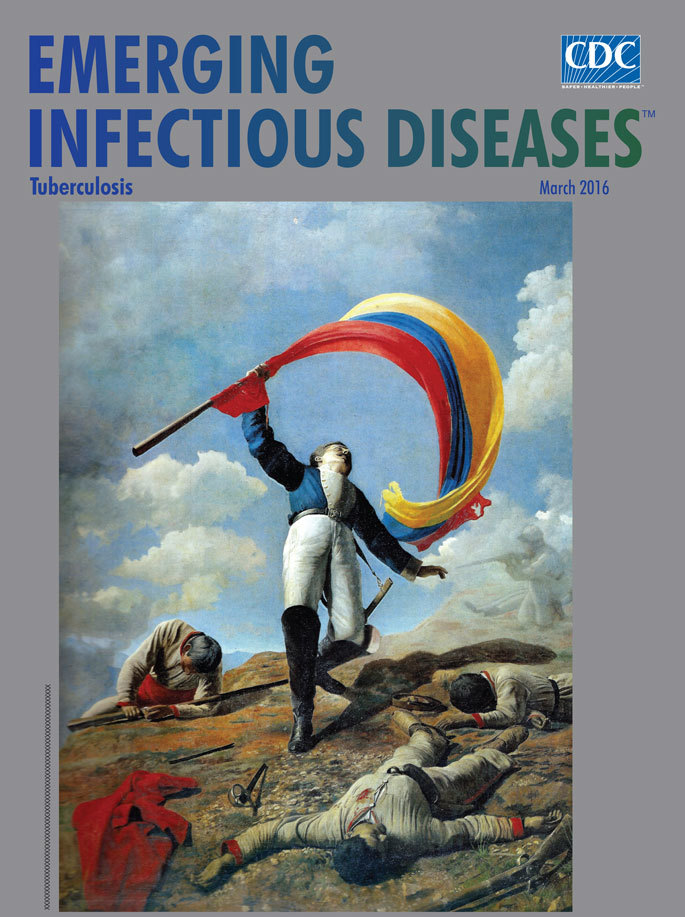
**Cristóbal Rojas (1857−1890), *La muerte de Girardot en Bárbula* (The death of Girardot in Bárbula), 1883. Oil on canvas, 113.9 in × 85.4 in/287 cm × 217 cm. **Public domain digital image (copyright expired)

Venezuelan artist Cristóbal Rojas was born in Cúa in the Valles de Tuy, a town that was war-torn throughout his early childhood. His grandfather, José Luis Rojas, taught the child how to draw, but when Cristóbal was 13, his father died, and he began work in a tobacco factory to help support his family. In 1878, an earthquake destroyed much of the Valles del Tuy region, and Rojas moved to Caracas, where he continued his artistic development under José Manuel Maucó at the Universidad Central de Venezuela. From 1880 through 1882, Rojas developed an interest in oil painting, and he captured memories of the impact of the 1878 earthquake in some of his early paintings. In Caracas, he also served as the assistant to the now comparably well-known artist Antonio Herrera Toro and worked with him on painting the interior of the Caracas Cathedral.

In 1883, Cristóbal Rojas exhibited one of his signature works, *La muerte de Girardot en Bárbula* (The death of Girardot in Bárbula), featured as this month’s cover image. He entered this painting into competition in the Salón del Centenario in Caracas, to commemorate the centennial of the birth of Simon Bolivar on whose side Rojas’ great grandfather had fought in the War for Independence; the painting remains his only heroic or patriotic work. This dramatic painting depicts an event in 1813 when the Colombian hero, Anastasio Girardot, a close confidant and supporter of Bolivar, was defeated by Spanish forces during the battle of Bárbula. Rojas shows the mortally wounded colonel falling backward as the flag curls down around him dominates the scene. Flanked by his fallen comrades, the stricken Girardot collapses, struck by a fatal shot as he attempts to raise the flag of the Republic. The painting was immediately purchased by the Government of Venezuela, and its execution won Rojas a medal and a scholarship from the government to study in France.

Rojas moved to Paris in late 1883. There he lived in Montmartre, spent much of his time studying in the Louvre, and began experimenting with different stylistic approaches and elements, from neo-Classical to Romanticism to Impressionism. Among his greatest works are renowned melancholic masterpieces that reflect suffering and early death, often from tuberculosis (TB), which was more common in that period. These works include *La miseria* (Misery, 1886, a desolate scene of a young husband sitting next to his supine wife who has just died in an impoverished setting) and *La primera y última comunión* (The first and last communion, 1888, a haunting scene of a priest administering the sacrament for the first time to a young girl who is dying in her mother’s arms). Art historian Vivian Barclay has remarked that whereas Rojas’ “Venezuelan audience wanted to see heroism and patriotism ... his Parisian audience wanted sadness and melodrama.” Having TB himself, Rojas returned to Venezuela from France, and cognizant of his own impending death, he completed his final work, *El purgatorio*, a depiction of purgatory. He began the painting while in France but completed it in Venezuela in 1890. At the end of that year, when Rojas died of TB at age 32, nearly one third (30%) of all deaths in Paris were attributed to TB.

In France and Venezuela, the incidence of TB was much higher in the 19th century than it is today. However, actual incidence numbers are crude estimates, as it was not until 24 March 1882—a year before the exhibition of *La muerte de Girardot en Bárbula*—that Robert Koch identified the tubercle bacillus as the etiologic agent. In Venezuela today, the average national incidence of TB is moderate, more than 27 cases per 100,000 persons. Despite recent advances in TB care and control that have helped lower TB incidence in Venezuela, great disparities exist in the incidence of TB across different segments of the population, revealing inequities that are prominent in most Latin American countries. Disability and death due to TB continue to have major economic and social implications for areas of high endemicity, where economic and disease issues take on a chicken-and-egg type of relationship.

Although TB was romanticized in the literature and music of the 19th century, a few studies from the pre-antimicrobial era enable us to estimate that the lifetime case-fatality rate of smear-positive pulmonary cases was more than 70%. Today, globally, TB is responsible for more than 9 million new cases of active disease and 1.5 million deaths annually, or a death rate of more than 16%. However, since the 1990s, the emergence of multidrug resistance (MDR) in *Mycobacterium tuberculosis*, principally in the developing world and in the former Soviet Union, is reflected now in an estimated 480,000 incident MDR TB cases per year, of which only about a quarter are detected and reported. Case-fatality rates among the growing population of patients with MDR TB approximate those of TB in general seen in the pre-antimicrobial era (the time of Cristóbal Rojas’ death). If drug resistance increases substantially, TB elimination will become more difficult to achieve. To regain the lost ground, it is important that health professionals engage in informed and timely approaches to diagnosis, treatment, and prevention and that there be expansion of testing for TB drug susceptibility and HIV, provision of antiretroviral treatment, reevaluation of existing drugs for their anti-TB potential, and development of a greater number and variety of antimicrobials targeting TB.
